# A neglected problem of developing countries: Noncystic fibrosis bronchiectasis

**DOI:** 10.4103/1817-1737.44781

**Published:** 2009

**Authors:** Arzu Babayigit, Duygu Olmez, Nevin Uzuner, Handan Cakmakci, Tuba Tuncel, Ozkan Karaman

**Affiliations:** *Department of Pediatric Allergy, Dokuz Eylul University Medical School, Balcova, 35340, Izmir, Turkey*; 1 *Department of Radiology, Dokuz Eylul University Medical School, Balcova, 35340, Izmir, Turkey*

**Keywords:** Children, etiology, noncystic fibrosis bronchiectasis

## Abstract

**BACKGROUND:**

Bronchiectasis has been defined as the abnormal and permanent dilation of the bronchi. It is still an important problem in many developing countries.

**AIM:**

The aim of this study was to identify the chacteristics and underlying etiology of children followed with the diagnosis of noncystic fibrosis bronchiectasis.

**MATERIALS AND METHODS:**

Children with bronchiectasis confirmed with high-resolution computed tomography were enrolled into the study. The data of the patients, including symptoms of the disease, age at the onset of symptoms, findings of physical examination, labrotory investigations performed in order to identify the etiology of bronchiectasis, etiology of bronchiectasis if found, radiologic findings and treatment modalities were noted.

**RESULTS:**

Sixty-six children between 1 and 17 years were included in the study retrospectively. Forty-four of them were males (66.7%) and 22 (33.3%) were females. The most common presenting symptoms were cough (100%) and sputum expectoration (50%). An underlying etiology was identified in 44 (66.7%) of the study subjects. The four most common underlying causes were found as infections (21.2%), asthma (16.7%), aspiration syndromes and/or gastroesophageal reflux disease (9.1%) and immunodeficiency syndromes (7.6%), respectively.

**CONCLUSION:**

Identifying an underlying etiology will have a significant effect on the management of noncystic fibrosis bronchiectasis. Defining the cause of bronchiectasis may also decrease its incidence, progression and complications.

Bronchiectasis is a progressive condition characterized by irreversible dilatation of the airways. This disease is considered as an orphan either because it is rare or because it is more prevalent in developing countries than in the developed world. Bronchiectasis unrelated to cystic fibrosis was termed an “orphan disease”.[1,2] The prevalence of bronchiectasis is not well characterized. Altough improved sanitation and nutrition, introduction of childhood immunization, particularly against pertussis and measles, and the early and frequent use of antibiotics resulted in a decline in the prevalence of bronchiectasis,[3] the disease is still a problem in developing countries like our country, Turkey.[4–6] As bronchiectasis is now rare in many developed countries, this has lead to limited research and treatment development on this condition. Over the past years, there have been relatively few studies that have assessed the clinical features of bronchiectasis in childhood.

The aim of this study was to determine the characteristics of children with noncystic fibrosis bronchiectasis and to evaluate the etiologic factors that cause the disease.

## Materials and Methods

Children followed with the diagnosis of bronchiectasis at the Dokuz Eylul University Hospital Pediatric Allergy Department in Izmir, Turkey, between 2003 and 2008 were included in the study retrospectively. The diagnosis of bronchiectasis was confirmed radiologically by high-resolution computed tomography (HRCT) for every patient. The following information was found from the patient's file: age at the onset of symptoms, symptoms of the disease such as sputum production, cough, hemoptysis, failure to thrive, history of childhood respiratory infections, history of recurrent wheezing and family history of chronic respiratory disorders. The findings of physical examination, including oscultation findings and presence of clubbing and chest deformity, were also noted. Because HRCT is accepted as the gold standard for diagnosis of bronchiectasis, all patients in the study group were selected from children in whom the diagnosis was confirmed by HRCT. Sweat chloride tests were performed in all patients in order to exclude cystic fibrosis. In the selected patients, genetic mutation analysis for cystic fibrosis was also performed. Serum levels of immunoglobulin (Ig) A, subgroups and total G, M, E and alfa-1 antitrypsine were obtained. All patients aged more than 6 years performed pulmonary function tests with spirometry. Flexible fiberoptic broncoscopy was performed in selected cases (15 of the patients) in whom the etiology of bronchiectasis could not be found in order to exclude foreign body aspiration and to obtain bronchoalveolar lavage fluide for culture. Foreign body aspiration was not detected in our study group.

Patients who had symptoms of swallowing problems and gastroesophageal reflux were investigated by barium fluoroscopy or/and gastroesophagial reflux scintiscans.

Primary ciliary dyskinesia was diagnosed with history of recurrent sinusitis, otitis, pneumonias, presence of situs inversus totalis in two patients, and their ciliary ultrastructures were also evaulated by electron microscopy on nasal epithelial biopsies.

Patients were followed-up with medical treatments including antibiotic use in exacerbations, mucolytics, bronchodilators and chest physiotherapy. Inhaled steroids were given to patients who had bronchial hyperreactivity and positive bronchodilator reversibility. Surgery (lobectomy) was performed in only four patients who had recurrent lower respiratory tract infections and localized disease.

### Statistical analysis

The SPSS Inc., 233 S. Wacker Drive, 11^th^ Floor, Chicago, IL 60606-6307, 11.0 statistical package for Windows was used throughout the study. Results were shown as mean±SD.

## Results

Sixty-six children who had diagnosis of bronchiectasis confirmed with HRCT were retrospectively included in our study. Forty-four of them were males (66.7 %) and 22 (33.3 %) of them were females. Mean age of the patients was 9.20 ± 4.38 years (range 1-17 years) at presentation and they had been symptomatic for 4.30 ± 4.14 years (range 0.5-15 years). The most common presenting symptom was cough (100%) followed by sputum expectoration (50%). Hemoptysis history was detected in only three (4.5%) of our patients. Symptom history at the time of diagnosis in our study group is given in Table 1.

**Table 1:** Symptoms at the time of diagnosis

Symptom history	Number of cases	%
Cough	66	100
Sputum	33	50
Recurrent pneumonia	26	39.4
Recurrent wheezing	13	19.7
Hemoptysis	3	4.5
Failure to thrive	18	27.3

On physical examination, cracles were detected in 72.7% and ronchi in 15.1% of the children. Clubbing was present in 22.7% of the study group. The physical examination findings of our study population are summarized in Table 2.

**Table 2:** Findings of physical examinations at the time of diagnosis

Findings	Number of cases	%
Crackles	48	72.7
Ronchi	10	15.1
Clubbing	15	22.7
Pectus carinatum	3	4.5
Scoliosis	2	3
Normal	2	3

Forty-two (63.6%) of the children were able to do pulmonary function tests. 85.7% of them had abnormal function tests. Fourteen patients (33.3%) had obstructive lung changes, five (11.9%) had restrictive lung changes and 17 (40.5%) had combined obstructive and restrictive lung changes.

An underlying etiology was identified in 44 (66.7%) of our patients [Table 3]. The four most common underlying etiologies in our patients were found as infections (21.2%), asthma (16.7%), aspiration syndromes (9.1%) and immunodeficiency syndromes (7.6%), respectively. In five immunodeficient children with bronchiectasis, deficiency of Ig A, deficiency of Ig G2, deficiency of both IgA and IgG2, deficiency of Ig M and common variable immune deficiency were detected, respectively.

**Table 3:** Identified causes of bronchiectasis

Etiological factors for bronchiectasis	Number of patients	%
Infection	14	21.2
Bacterial pneumonia	8	12.1
Adenovirus	2	3
Varicella	1	1.5
Tuberculosis	3	4.6
Asthma	11	16.7
Aspiration syndromes/gastroesophageal
reflux disease	6	9.1
Immune deficiency (antibody deficiency syndromes)	5	7.6
Primary ciliary dyskinesia	4	6.1
Chest deformity (severe scoliosis)	2	3
Congenital structural abnormalities	2	3
Vascular ring	1	1.5
Congenital lobar emphysema	1	1.5
Unknown	22	33.3
Total	66	100

In the radiologic findings of the patients (CT findings), 42 children (63.6%) had only one affected lobe, most commonly the left lower lobe (42.4%) [Table 4]. Thirteen of the cases (19.7%) had two lobes involved, whereas 11 patients (16.7%) had three or more lobes involved.

**Table 4:** Localization of bronchiectasis in the study group

Localization	%
Left lower lobe	42.4
Right middle lobe	25.8
Lingula	25.8
Right lower lobe	22.7
Right upper lobe	13.6
Left upper lobe	7.6
Bilateral diffuse	12.1

The treatment modalities used by children include bronchodilators (16.7%), mucolytics (24.2%), inhaled steroids (25.2%), combination of bronchodilators and mucolytics (16.7%) and chest physiotherapy (50%). Ten of the patients (15.2%) were not using any medical treatments. Surgery (lobectomy) was performed in only four patients (6%) who had recurrent pneumonias and localized disease. One of these patients who had lobectomy performed also had a history of recurrent hemoptysis.

## Discussion

Bronchiectasis was first described as “production of fetid sputum along with bronchial dilatation”.[7] It is a pathological description of a disease process that has many possible causes. In a previous large series, the majority of the cases were due to extrinsic factors, especially childhood respiratory infections, as the cause of permanent bronchial damage.[8] In a study by *Eastham et al*,[9] previous respiratory tract infection was found as the most common cause of 93 noncystic fibrosis bronchiectasis cases. Immunodeficiency and bronchiolitis oblierans were the second and third most common reported causes in this study.[9] Karakoc *et al*, evaulated 23 children with bronchiectasis from Adana, Turkey, and they reported that infections, cystic fibrosis and immunodeficiency syndromes were the most common causes of bronchiectasis.[4] Karadag *et al*, evaluated 111 children and found postinfectious etiology and immunodeficiencies as the most common causes of noncystic fibrosis bronchiectasis.[5] The etiologic factors underlying childhood bronchiectasis were also investigated with another study from Ankara, and the authors of this study concluded that infections, asthma and primary immunodeficiency syndromes were the three most common causes of noncystic fibrosis bronchiectasis.[6] In the recent years, with early immunization and the widespread use of antibiotics in childhood, postinfectious etiology is likely to be less relevant, especially in developed countries. Based on this information, investigations of intrinsic defects or noninfective extrinsic causes that predispose to bronchial inflammation or infection in more developed areas can be suggested.[8] In our study, we found infections, asthma, aspiration syndromes and immunodeficiency syndromes as the most common causes of bronchiectasis in Izmir. Izmir is the third largest city of Turkey, which has a population of approximately 3.5 million people, located in the western part of the country. Although the socioeconomic status varies widely, most of the people live under hygienic conditions and childhood immunization programmes are well organized. We believe that the more urbanized features of the city of Izmir can explain the lower propotion of infectious etiology in our study group. Bronchial asthma was found as the second common cause of noncystic fibrosis bronchiectasis in this study. This may be due to the underdiagnosis of asthma in children with only cough who had no wheeze or pulmonary oscultation findings. We believe that untreated bronchial inflammation may lead to bronchiectasis in this group of patients. Aspiration syndromes and gastroesophageal reflux disease were found as the third common cause of bronchiectasis in the current study. In six patients (9.1%), aspiration syndromes and/or gastroesophageal reflux disease were detected as the cause of bronchiectasis in this study. Two of these patients were mentally retarded, and it was learnt that thay were hopitalized many times because of aspiration pneumonias. The remaining four children without neurological defects were diagnosed as with gastroesophageal reflux disease. There is evidence that gastroesophageal reflux disease in children without neurological defects is associated with a several-fold increase in the risk of sinusitis, laryngitis, asthma, pneumonia and also bronchiectasis.[10]

The majority of the clinical studies reported that bronchiectasis patients had symptoms in the preschool years.[7] In 74.2% of our study group, clinical symptoms had begun before the age of 5 years. Cough and sputum production were the major symptoms in our study, which is consistent with other reports in the literature.[3,7] Hemoptysis is a frequent symptom in adult bronchiectasis patients, but it is relatively uncommon in children.[11] Only three patients (4.5 %) in our study group suffered from hemoptysis. One of these patients who had recurrent massive hemoptysis and pneumonias had undergone surgery.

In the diagnosis of bronchiectasis, chest radiographs usually have nonspecific findings of increased lung markings.[12] HRCT has proven to be a reliable and noninvasive method for the assessment of bronchiectasis.[13,14] HRCT can accurately diagnose bronchiectasis, localize and describe areas of parenchymal and bronchiolar abnormality and also mucus plugging.[14,15] HRCT scanning is the method used in all cases in our study group in order to diagnose bronchiectasis.

Bronchiectasis is most commonly seen in the lower lobes, especially in the left lower lobe. The upper lobes are affected less frequently, except in cystic fibrosis, probably because of the faciliated mucociliary clearance by gravity.[4,6] In our study group, after the left lower lobe (42.4%), right middle lobe (25.8%), lingula (25.8%) and right lower lobes (22.7%) were the the other more affected three lobes, respectively.

In conclusion, we emphasized that identification of the underlying cause is the most important step in the treatment and prevention of bronchiectasis. Earlier diagnosis of even milder disease will also change the outcomes and decrease the morbidity and mortality of disease. Research needs urgently to be encouraged and bronchiectasis should not be an orphan any more. We were able to determine the etiology of bronchiectasis in 66.7% of our patients and infections, asthma, aspiration and immunodeficiency syndromes were found to be the most common causes of noncystic fibrosis bronchiectasis in our study population.
